# Leadership in ophthalmological services

**DOI:** 10.22336/rjo.2021.42

**Published:** 2021

**Authors:** Consuela-Mădălina Gheorghe

**Affiliations:** * Philologist, Authorized translator

Although it is not clearly defined, the term “leadership” has some generally accepted characteristics such as: the ability to create a clearer vision, the ability to share the vision with the others and to motivate them to follow it, the capacity to offer organized structure, the instruments and the information that allow the others to be successful in following the vision and approaching the risks and conflicts that arise during the following of that specific vision. 

Leadership is the process of social influence through which an individual leads a group towards achieving the objectives. Leadership does not only involve convincing other individuals do something, but also the ability to change the attitude of the members of the group, to mobilize and train them to achieve common goals. The action of leading is characterized between the social activities by the possibility of exerting a certain influence over a group.

There are two types of leadership: traditional and effective, each having its own characteristics and implying a certain set of attributes. Traditional leadership assumes that the individual, the leader, has a position on a hierarchy and title, his decision being the one that is embraced and followed by all the members of the group. Effective leadership implies that the activity of the leader is highly connected with the group, so the decisions are taken at the group level, his role being the one of listener and “instrument” through which the group members reach their highest potential.

In ophthalmology, literature on leadership development programs is scarce. Such programs are important means for the cultivation of leadership talent. Leadership in ophthalmology is a must in many contexts: clinical, surgical, at institutional level, in healthcare organizations, research, education, etc. Although there is a need for leadership, the curricula mainly focus on clinical skills to the detriment of teaching and assessing non-clinical competencies. The result is that fewer ophthalmologists feel confident to assume leadership roles. At the same time, it is needless to mention that it takes years of training to become a competent leader. Ophthalmic leaders should have specific skills, according to the context in which they are working.

When dealing with leadership in ophthalmology it is worth mentioning the concept of Ophthalmic World Leaders. 

According to Cynthia Matossian, MD, FACS, the Founder and Medical Director of Matossian Eye Associates, an integrated ophthalmology and optometry private practice with locations in New Jersey and Pennsylvania, OWL originally stood for Ophthalmic Women Leaders but the acronym has recently changed to Ophthalmic World Leaders to be more inclusive. Its mission is to encourage diversity in leaders, to promote and develop leadership, to advance ophthalmic innovation and patient care. OWL has a presence at major ophthalmic conferences; this is expanding as the organization becomes more recognized. They create networking opportunities for industry and physicians at regional and national meetings. In addition, they host events on leadership, negotiations, communications, etc. (https://www.researchgate.net/publication/1GV9Jm2u7rmsCe65wKzPTw5jtS38n2tVEGigy). 

In the article entitled “Developing leadership skills in young ophthalmologists”, the authors, Green C, Atik A and Hay M stated that in the era of continuous changes, there is an increased need to determine the ophthalmologists who have leadership skills to be at the forefront of change. Even if the need for ophthalmic leadership has been recognized, there are very few leadership skills as a component of their core curricula, focusing on clinical knowledge with less emphasis on teaching of non-clinical professional competencies. In ophthalmology, effective leadership reflects in organizations better run, challenges that are addressed holistically by ophthalmologists, etc. 

At the same time, effective leadership is reflected in increased patient satisfaction and the specialty literature acknowledges that the most effective individuals in organizations are both managers and leaders. However, a distinction should be made between the two, as they are not one and the same. According to Catherine Green, Alp Atik and Margaret Hay, who published the review article entitled “Developing leadership skills in young ophthalmologists”, management skills can be systematically learned and can provide structure for new leaders to plan their development. In addition, whilst leaders have management competencies, leadership itself extends beyond the competencies of management alone. 

In conclusion, in order to be an effective leader, it is important to have a constant focus and be a good listener, as it essential to convince as many people as possible to pursue your goals, which will inevitably become their goals too.

